# Antibiotic stress affects the secretion and physicochemical features of extracellular vesicles produced by *Helicobacter pylori*

**DOI:** 10.1093/jac/dkaf172

**Published:** 2025-05-29

**Authors:** Paweł Krzyżek, Agnieszka Opalińska, Paweł Migdał, Kaja Tusiewicz, Paweł Szpot, Marcin Zawadzki, Barbara Krzyżanowska, Michał Jerzy Kulus, Marzenna Podhorska-Okołów, Beata Sobieszczańska

**Affiliations:** Department of Microbiology, Faculty of Medicine, Wroclaw Medical University, Wroclaw, Poland; Laboratory of Nanostructures, Institute of High Pressure Physics, Polish Academy of Sciences, Warsaw, Poland; Department of Bees Breeding, Institute of Animal Husbandry, Wroclaw University of Environmental and Life Sciences, Wroclaw, Poland; Department of Forensic Medicine, Wroclaw Medical University, Wroclaw, Poland; Department of Forensic Medicine, Wroclaw Medical University, Wroclaw, Poland; Department of Social Sciences and Infectious Diseases, Faculty of Medicine, Wroclaw University of Science and Technology, Wroclaw, Poland; Department of Microbiology, Faculty of Medicine, Wroclaw Medical University, Wroclaw, Poland; Division of Ultrastructure Research, Wroclaw Medical University, Wroclaw, Poland; Division of Ultrastructure Research, Wroclaw Medical University, Wroclaw, Poland; Department of Physiotherapy, University School of Physical Education, Wroclaw, Poland; Department of Microbiology, Faculty of Medicine, Wroclaw Medical University, Wroclaw, Poland

## Abstract

**Background:**

Bacterial extracellular vesicles (EVs) may reduce the effectiveness of various antimicrobials; however, the impact of antibiotics on the secretion and properties of EVs produced by *Helicobacter pylori* has not been established.

**Methods:**

Using clinical *H. pylori* strains and culture in EV-depleted media, the influence of ¼ × MIC of clarithromycin, metronidazole and levofloxacin on EV features was determined. Physicochemical properties of EVs were measured using nanoparticle tracking analysis and dynamic light scattering. Determination of fatty acid profiles of EVs and bacterial cells was performed with GC triple-quadrupole tandem MS. Bacteria and EVs were observed by scanning and transmission electron microscopy, respectively.

**Results:**

Antibiotic stress induced in *H. pylori* affects the secretion intensity and physicochemical features of EVs secreted by this bacterium in a strain- and antibiotic-dependent manner. Exposure to ¼ × MIC of metronidazole or levofloxacin increased the secretion of EVs and contributed to significant changes in their fatty acid profile, whereas treatment with ¼ × MIC of clarithromycin did not induce such changes. Regardless of the culture conditions and the strain analysed, the existence of a conservative process of selective packaging of C17:0 fatty acids into EVs and a substantial limitation of this phenomenon for C14:0, C18:1 and C19c:0 was demonstrated.

**Conclusion:**

This is the first study showing the modulatory effect of antibiotic stress on the secretion and physicochemical features of EVs produced by *H. pylori*, as well as the first to suggest the involvement of EVs in maintaining the appropriate membrane fatty acid composition of this bacterium.

## Introduction

*Helicobacter pylori* is a spiral-shaped Gram-negative bacterium with the unique ability to thrive in an acidic stomach environment, thanks to the large amount of urease produced.^1,2^ This characteristic is further exaggerated by the vast arsenal of toxins, lytic enzymes and adhesins, all of which are responsible for inducing chronic gastritis.^1,3,4^ Moreover, the chronic progressive inflammatory response against *H. pylori* may eventually lead to long-term health implications, such as peptic ulcer disease, gastric cancer and mucosa-associated lymphatic tissue lymphoma.^5,6^ Since more than half of the world’s human population harbours *H. pylori*, its eradication is a significant challenge, hampered by the alarming rates of *H. pylori* resistance.^7^ According to the WHO’s comprehensive assessment, the global *H. pylori* resistance to three antibiotics, namely clarithromycin, metronidazole and levofloxacin, has surpassed the 15% threshold.^8^ This trend is a serious concern for public health and clinical providers.^9,10^

Routine diagnostic methods and strategies restricting therapeutic failures of *H. pylori* infections are almost exclusively focused on chromosomally encoded genetic changes. Point mutations in the drug target sites are the most studied mechanism of antibiotic resistance in *H. pylori* and involve modifications in the following genes: *23S rRNA* (A2142G/C, A2143G, A2144T, T2717C and C2694A) for clarithromycin resistance, *gyrA* (N87K/L/I/A and D91G/N/A/Y/H) for levofloxacin resistance, and *rdxA* (R16H/C, Y47C, A67V/T and V204I) for metronidazole resistance.^11–14^ Despite the key role of genetic mutations in the spread of antibiotic resistance, evidence for the importance of other survival strategies is growing rapidly.^15^ Tolerance to antibiotics often allows microorganisms to overcome exposure to antimicrobials.^16^ Strikingly, this phenomenon may not only prolong treatment and cause recurrence of infections, but also increase the risk for the development of antibiotic resistance.^16,17^ Whereas the role of coccoid forms or biofilm formation in the context of therapeutic failures of *H. pylori* infections is documented,^18–21^ the importance of other processes, including the secretion of extracellular vesicles (EVs), is still ignored.

Bacterial EVs are nanometric particles that are released from cells and are delimited by a lipid bilayer and cannot reproduce independently.^22^ Several decades ago, the biological function of these structures was poorly understood; however, various recent genetic and biochemical studies have established that the production of EVs by microorganisms is an active process and should be considered as a vital secretion system.^23,24^ The benefits of producing EVs include the export of toxic metabolites and antimicrobial substances, protection against the immune system, spreading of resistance genes, participation in maintaining the biofilm structure and transport of virulence factors.^23,25^ As detailed reviews describing EVs of *H. pylori* show, most research investigating these structures is focused on their cytotoxic activity and participation in the development of various diseases.^26–30^ To our knowledge, there is only a single original article reporting the involvement of EVs in tolerance of *H. pylori* against antibiotics,^31^ and despite its value, it has not verified the ability of antibiotics to modulate parameters of these structures.

Therefore, this original work aimed to determine the impact of sublethal concentrations of antibiotics on the secretion capacity and physicochemical features of EVs produced by *H. pylori*.

## Materials and methods

### Storage and revival of bacterial strains

The research was conducted using three clinical *H. pylori* strains (1CML, 2CML, 3CML), characterized by our team in previous studies.^32,33^ The strains were stored at −80°C in Tryptic Soy Broth (TSB; Oxoid, France) with 30% glycerol. To revive them, the strains were plated onto Columbia agar (Difco, Poland) supplemented with 10% horse blood (Graso Biotech, Poland) and incubated for 3 days at 37°C in microaerophilic conditions (GENbox microaer kits; bioMérieux, France). After incubation, the bacteria were subcultured onto fresh solid media once again.

### Impact of the serum on bacteria

The effect of the culture media composition on the bacterial physiology was assessed using 24-well ventilated titration plates (Bionovo, Poland) filled with Brain-Heart Infusion broth (BHI; Oxoid) and different concentrations (0%, 2%, 5% and 10%) of FCS (Gibco, UK) and EV-depleted FCS (FCS^-EVs^; Gibco). The wells of titration plates were loaded with a final volume of 1.5 mL of the tested culture medium and a bacterial density of 10^8^ cfu/mL. The plates were subjected to a 3 day incubation at 37°C under microaerophilic conditions and shaking at 100 rpm. After incubation, bacterial multiplication and biofilm formation were estimated with the preparation of samples as previously described.^34^ The bacterial multiplication was evaluated spectrophotometrically, and the biofilm formation was determined using a crystal violet staining assay and spectrophotometry. The absorbance of planktonic cells and biofilms was measured using an Asys UVM 340 microplate reader (Biochrom Ltd, UK) at OD_600_ and at OD_590_, respectively. The values of negative controls (culture medium without bacteria) were subtracted from the measurements of the experimental samples. For both tested parameters three biological repetitions were performed (*n* = 3).

### Impact of antibiotic stress on bacteria

The MICs of clarithromycin, metronidazole and levofloxacin (all from Merck) for *H. pylori* strains grown in BHI + 10% FCS^-EVs^ were determined using the broth microdilution method.^33^ Each well of the 24-well titration plates was filled with 1.5 mL of culture medium, bacteria (10^8^ cfu/mL) and a concentration gradient of one of the tested antibiotics (1–512 mg/L). A well containing bacterial suspension without antibiotic, and one containing pure culture medium without bacteria, served as positive and negative controls, respectively. The plates were cultured for 3 days at 37°C under microaerophilic conditions and shaking at 100 rpm. The well containing the lowest antibiotic concentration in which lack of turbidity was observed was defined as the MIC. For ¼ × MIC of each antibiotic, the effect on bacterial multiplication and biofilm formation was additionally determined, with the methodology described above. The tests were performed in three biological replicates (*n* = 3). The ultrastructure of 3 day planktonic cells exposed to ¼ × MIC of each tested antibiotic or non-exposed (a positive control) was examined using scanning electron microscopy (SEM) with the preparation of samples as previously described.^32,33^ Briefly, bacterial pellets were fixed using 0.5 mL of 2.5% glutaraldehyde solution (Merck), rinsed three times with 0.1 M cacodylate solution (Merck) and subjected to a graded alcohol series. The fixed samples were coated with a layer of carbon using an EM ACE600 sputter (Leica Microsystems, Germany) and analysed with SEM (Auriga 60, Germany).

### Isolation and physicochemical properties of EVs

The polymer-based isolation of EVs was performed using the Total Exosome Isolation kit (from cell culture media; Thermo Scientific, Waltham, MA, USA) according to the manufacturer's protocol. Briefly, bacterial culture was subjected to centrifugation at 2000 × **g** for 30 min, after which the obtained supernatant was passed through a 0.45 µm cellulose acetate filter (Corning, New York, USA). The supernatant was mixed with the kit reagent (2:1), vortexed, and stored overnight at 4°C. Then, the samples were centrifuged at 10 000 × **g** for 60 min, the supernatant was discarded, and the EV pellet was suspended in PBS. To confirm absence of contaminating bacterial cells, 10 µL aliquots of the obtained solutions were spotted on Columbia agar + 10% horse blood and incubated for 7 days at 37°C under microaerophilic conditions. Solutions with EVs were stored at −80°C until analysis.

The average size, size distribution and concentration of EVs were determined by nanoparticle tracking analysis using the NanoSight NS500 instrument (Malvern Instruments, UK).^35^ The light source used was a blue diode laser with a wavelength of 405 nm. The tests were conducted at an ambient temperature of 23°C ± 0.4°C. Each biological replicate underwent nine independent measurements and was expressed as the mean of these values. For each tested parameter three biological repetitions were performed (*n* = 3). If necessary, samples were diluted with PBS when the threshold for measuring the concentration of nanoparticles was obtained. For such samples the dilution factor was additionally considered in the final calculation of the EV concentration. To acquire information about the ability of the tested strain to secrete EVs, the concentration values were normalized to the OD_600_ of extracted cultures.^36,37^

The zeta potential of EVs was assessed using the Zetasizer Nano-ZS ZEN 3600 analyser (Malvern Instruments).^38,39^ The setup included a laser Doppler electrophoresis analyser with a wavelength of 633 nm. The tests were performed in DTS1070 cuvettes (Malvern Instruments) with an automatic analysis model and an ambient temperature of 25°C. Each biological replicate underwent six independent measurements and was expressed as the mean of those values. Tests were performed in three biological repetitions (*n* = 3).

The ultrastructural quality of EVs was examined using transmission electron microscopy (TEM) with the preparation of samples as previously described.^35^ Briefly, PBS-suspended EV solutions were placed onto Formvar-carbon-coated copper grids and fixed in 2% paraformaldehyde (Thermo Scientific) and 2.5% glutaraldehyde solution (Serva Electrophoresis, Heidelberg, Germany). Next, the grids were counterstained in Uranyless (Electron Microscopy Sciences, Hatfield, PA, USA) and a 3% lead citrate solution (Electron Microscopy Sciences). Finally, the grids were embedded in 0.13% methyl cellulose (viscosity 25 cP; Merck). The grids were examined using TEM (JEM-1011, Jeol, Japan) at an accelerating voltage of 80 kV.

### Comparative analysis of the fatty acid profile of bacteria and EVs

Samples containing pelleted bacterial cells or EVs were held in 1.5 mL Eppendorf tubes filled with 200 µL of PBS. Into each Eppendorf tube, 10 µL of an internal standard (palmitic acid-d31 at a concentration of 10 mg/L; Merck) was added. After vortex-mixing, liquid-liquid extraction with 0.5 mL of isooctane (Merck) was carried out for 30 min in an ultrasound bath. The sample was then centrifuged at 13 500 rpm for 3 min. The organic phase was transferred into a 2 mL Eppendorf tube and evaporated to dryness under a stream of inert nitrogen gas at 40°C. The dry residues were dissolved in 50 µL of *N*-methyl-*N*-(trimethylsilyl)trifluoroacetamide solution (Merck) and the tube was then heated at 70°C for 20 min. After cooling, the solution was transferred into glass inserts of autosampler vials and analysed by GC triple-quadrupole tandem MS (Shimadzu, Japan).

The analytical equipment and method used were the same as in Krzyżek *et al*.^33^ All samples were analysed by MS first in scan mode (with an injection volume of 2 µL), and semi-quantitative analysis was performed in MRM mode (with an injection volume of 1 µL). The content of individual fatty acids in the analysed samples was determined by comparing the ratio of the signal intensity of the analyte to the signal intensity of the internal standard used. The results obtained for EVs were normalized to measurements of bacterial cells, and ratios of ≥2 or ≤0.5 were considered significant. For each tested parameter three biological repetitions were made (*n* = 3).

### Statistics

Statistical analyses were performed using the R program (R-3.4.4 for Windows; CRAN, Austria). The normality of data distribution was tested by the Shapiro–Wilk test. The Kruskal–Wallis test with Holm correction was applied for multiple comparisons to check differences between groups. To perform post hoc analysis, Dunn’s test with Bonferroni correction was used. For all the tests, a significance level of α = 0.05 was used.

## Results and discussion

### Selection of strains

The current research was conduced on three clinical MDR *H. pylori* strains characterized by us recently.^32,33^ These strains are equipped with the ability to adapt quickly to antibiotic stress, reflected in the modification of autoaggregation speed and biofilm formation.^33^ Importantly, the type of antibiotics used influences phenotypic features of biofilms. Keeping in mind that EVs possess the capacity to affect development of these structures,^40,41^ in the current research we decided to determine the impact of sublethal concentrations of antibiotics on the properties of EVs produced by these *H. pylori* strains.

### Bacterial response to the culture media composition and antibiotic stress

*H. pylori* is a microorganism with high nutritional requirements and its broth-based culture typically requires an addition of FCS (2%–10%).^42^ Such culture conditions have also been used in many studies analysing *H. pylori* EVs;^43–47^ however, in recent years it has been increasingly indicated that serum contains many animal-derived EVs.^48,49^ This in turn affects several parameters of the isolated microbial EVs (from the concentration to the size distribution).

**Figure 1.**
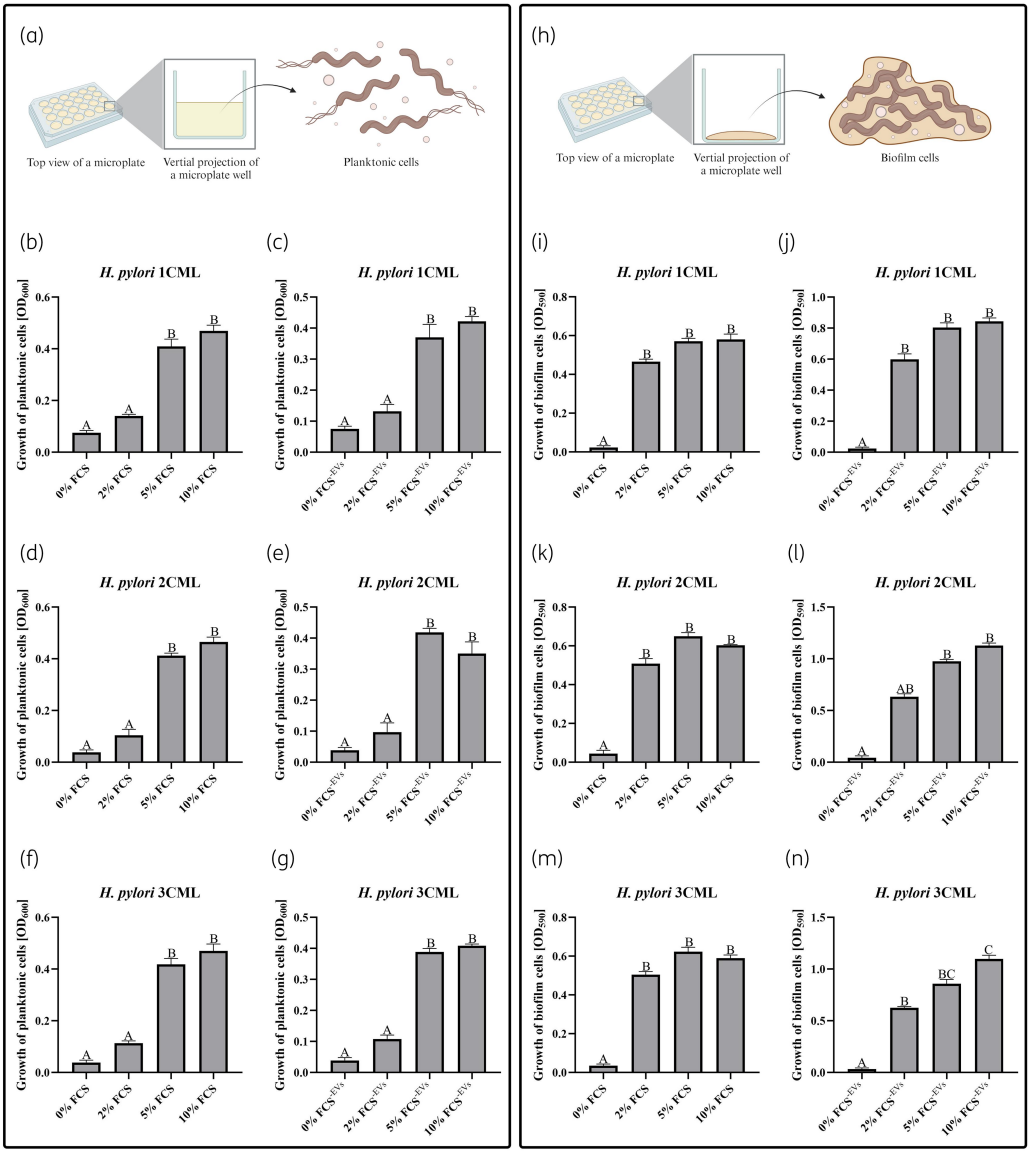
Assessment of physiological parameters of clinical *H. pylori* strains cultured in BHI supplemented with various concentrations of classic (FCS) and EV-depleted (FCS^-EVs^) serum. Bacteria were incubated in 24-well titration plates for 3 days at 37°C under microaerophilic conditions and shaking at 100 rpm. The growth of planktonic forms was evaluated spectrophotometrically, whereas the growth of biofilm forms was determined using a crystal violet staining assay and spectrophotometry. (a) Graphic showing the location of planktonic *H. pylori* forms in the titration plate. The optical density of planktonic forms of *H. pylori* 1CML (b, c), *H. pylori* 2CML (d, e) and *H. pylori* 3CML (f, g) strains is depicted. (h) Graphic showing the location of biofilm *H. pylori* forms in the titration plate. The optical density of biofilm forms of *H. pylori* 1CML (i, j), *H. pylori* 2CML (k, l) and *H. pylori* 3CML (m, n) strains is depicted. Each value is representative of three biological replicates (mean ± SEM). Values with different letters in a column are significantly different (*P* < 0.05, Kruskal–Wallis test with Holm correction). Graphics in parts (a) and (h) were created in bioRender (https://bioRender.com/c94r211).

In light of this, in the first stage of the study, we performed a comparative analysis of the density of planktonic and biofilm forms of *H. pylori* strains cultured in BHI supplemented with various concentrations of classic (FCS) and EV-depleted (FCS^-EVs^) serum. We noticed that in most cases, the increase in the serum concentration positively correlated with both examined bacterial parameters (Figure 1). Importantly, however, side-by-side analysis of the influence of the type of serum showed that whereas the density of planktonic forms in both variants was similar (Figure S1, available as Supplementary data at *JAC* Online), the biofilm development of *H. pylori* strains was significantly more promoted by FCS^-EVs^ (Figure S2). This phenomenon suggests that the type of serum in the culture medium may strongly influence the interpretation of the results and indicate the ability of animal-derived EVs to interfere with the development of biofilms, as was previously proposed.^50^ Regardless, we proved in our experiments that BHI + 10% FCS^-EVs^ has the most beneficial effect on the biofilm development of *H. pylori* and therefore we concluded that it would constitute an ideal medium for performing research on EVs secreted by this bacterium.

**Figure 2.**
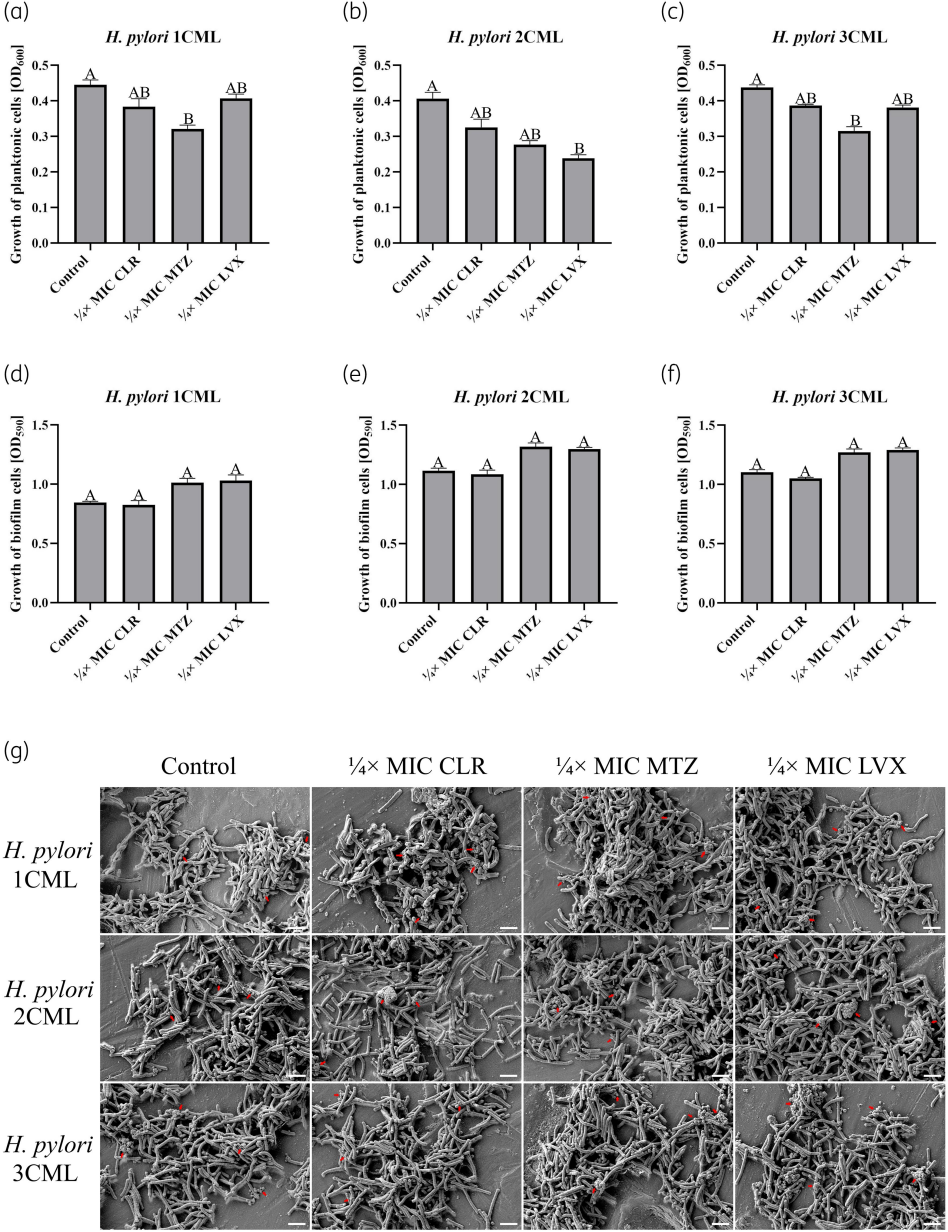
Assessment of physiological parameters of clinical *H. pylori* strains cultured in BHI + 10% FCS^-EVs^ under antibiotic stress. Bacteria were incubated in 24-well titration plates for 3 days at 37°C under microaerophilic conditions and shaking at 100 rpm. The growth of planktonic forms was evaluated spectrophotometrically, whereas the growth of biofilm forms was determined using a crystal violet staining assay and spectrophotometry. The parameters of *H. pylori* 1CML (a, d), *H. pylori* 2CML (b, e) and *H. pylori* 3CML (c, f) strains are depicted. Each value is representative of three biological replicates (mean ± SEM). The values with different letters in a column are significantly different (*P* < 0.05, Kruskal–Wallis test with Holm correction). (g) Photographs presenting the ultrastructure of *H. pylori* cells exposed to antibiotic stress. Red arrows mark representative locations of extracellular vesicles. Scale bars = 2 µm. CLR, clarithromycin; LVX, levofloxacin; MTZ, metronidazole.

After establishing the culture conditions, we assessed the impact of antibiotics on the physiological parameters of *H. pylori* strains incubated in BHI + 10% FCS^-EVs^. As done by us previously,^33^ we focused our experiments on three antibiotics for which an alarmingly high resistance of *H. pylori* is currently observed, i.e. clarithromycin, metronidazole and levofloxacin. After establishing concentrations of antibiotics necessary for use in experiments in BHI + 10% FCS^-EVs^ (Table S1), we noticed that in these conditions antibiotics affect the density of planktonic forms (Figure 2a–c), without a significant impact on the biofilm development (Figure 2d–f). Therefore, we decided that in further stages of EV secretion assessment we would normalize the concentration of these structures in relation to the optical density of the culture, following good practices recommended in other studies.^36,37^ Notably, SEM observations of the ultrastructure of *H. pylori* strains exposed to antibiotic stress allowed us to notice the presence of numerous EVs secreted by these bacteria (Figure 2g).

### Isolation of EVs and assessment of their physicochemical parameters

**Figure 3.**
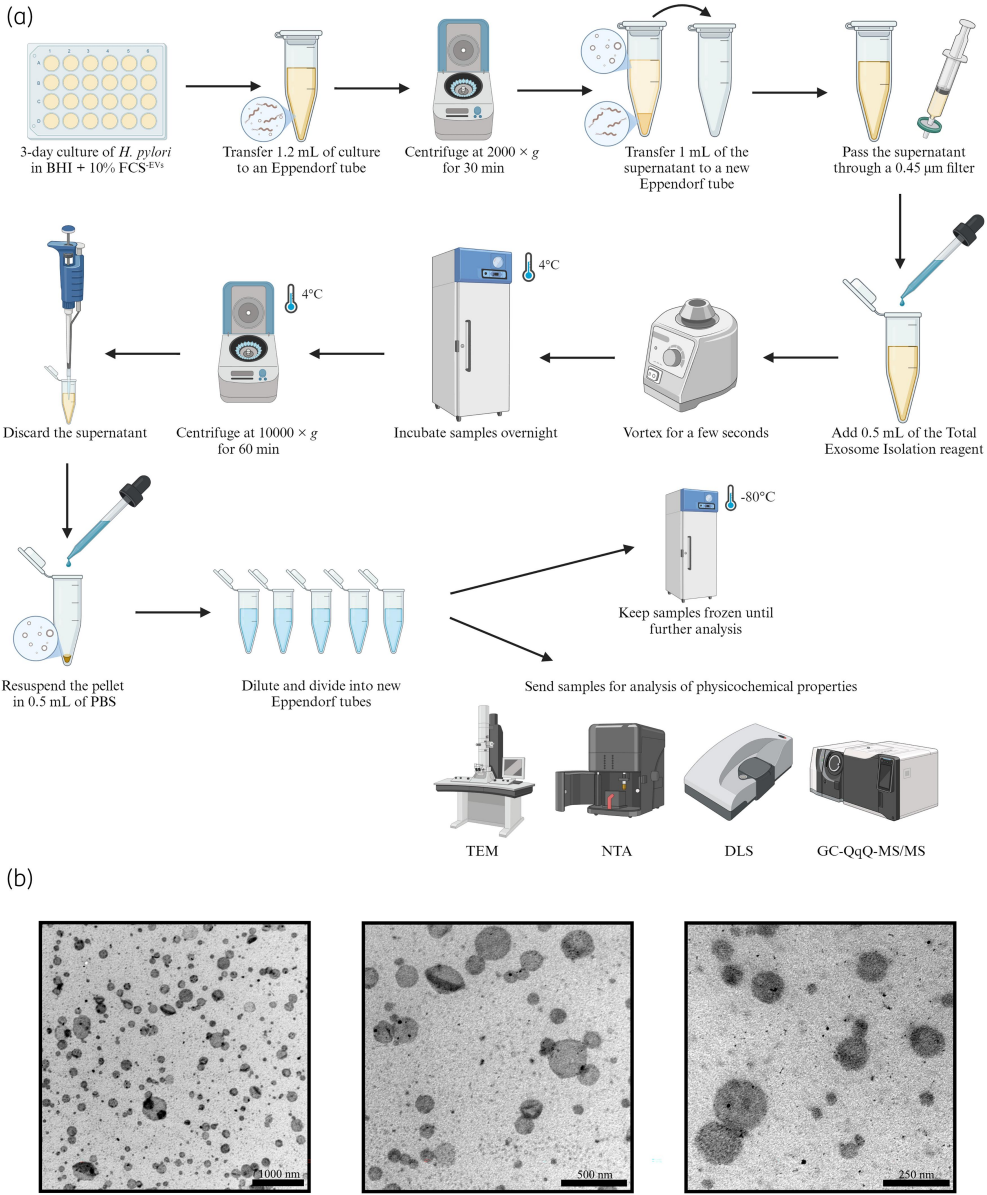
Isolation of *H. pylori* extracellular vesicles (EVs). (a) EV isolation procedure. (b) Representative ultrastructural photographs showing isolated EVs of *H. pylori*. DLS, dynamic light scattering; GC-QqQ-MS/MS, gas chromatography triple-quadrupole tandem mass spectrometry; NTA, nanoparticle tracking analysis; TEM, transmission electron microscopy. Part (a) was created in bioRender (https://BioRender.com/c92m596).

In the next stage, we were faced with the task of choosing an appropriate method for the isolation of EVs. As reviews describing this topic show,^51–53^ there is great methodological variation in this area. Ultracentrifugation is the most frequently used, although with the passage of time and the emergence of new techniques, its frequency has significantly decreased. In recent years, polymer precipitation has become the second most commonly applied technique. This technique uses superhydrophilic polymers to reduce the solubility of EVs, and unlike ultracentrifugation limits their deformation and allows for high-throughput processing of samples. In line with this, for the current set of our experiments, we chose polymer precipitation as the isolation method of EVs (Figure 3a). We confirmed the quality of these structures using TEM and observed the presence of numerous spherical EVs of *H. pylori* with dimensions typically in the range of 40–300 nm (Figure 3b). At the same time, microbiological structures that could contaminate these samples (e.g. flagellar fragments) were not detected.

**Figure 4.**
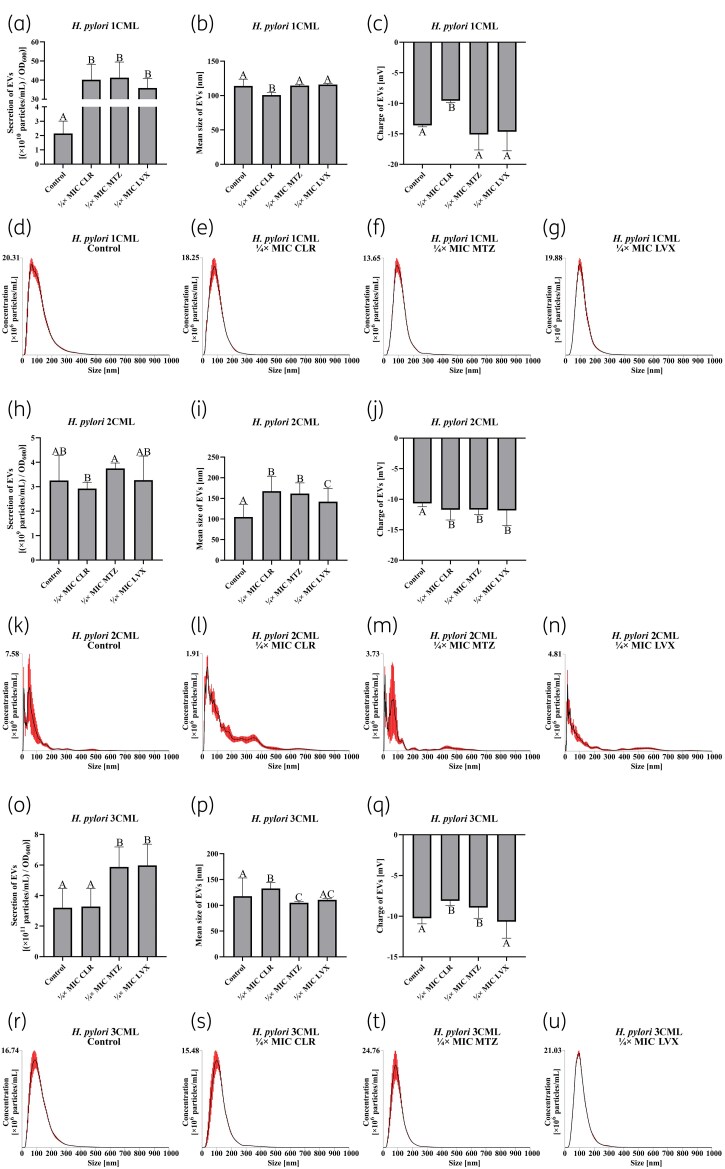
Assessment of physicochemical parameters of extracellular vesicles (EVs) isolated from clinical *H. pylori* strains cultured under antibiotic stress. Bacteria were incubated in BHI + 10% FCS^-EVs^ in 24-well titration plates for 3 days at 37°C under microaerophilic conditions and shaking at 100 rpm. The EV isolation procedure is shown in Figure 3. Depicted are: level of EV secretion (a), average EV size (b), electric charge of EVs (c) and size distribution charts of EVs (d–g) from *H. pylori* 1CML; level of EV secretion (h), average EV size (i), electric charge of EVs (j) and size distribution charts of EVs (k–n) from *H. pylori* 2CML; and level of EV secretion (o), average EV size (p), electric charge of EVs (q) and size distribution charts of EVs (r-u) from *H. pylori* 3CML. Each value is representative of three biological replicates (mean ± SEM). The values with different letters in a column are significantly different (*P* < 0.05, Kruskal–Wallis test with Holm correction). CLR, clarithromycin; LVX, levofloxacin; MTZ, metronidazole.

After validating the methodology of bacterial culture and isolation of EVs, in the next step we determined the impact of ¼ × MIC of antibiotics on the secretion and physicochemical parameters of EVs (Figure 4). We noticed that exposure to ¼ × MIC of metronidazole or levofloxacin induced EV secretion in two of the three strains examined (Figure 4a and o). This effect correlates well with findings with other microorganisms, linking the vesiculation-inducing activity of nitroimidazoles (e.g. metronidazole) and fluoroquinolones (e.g. levofloxacin) with their DNA-targeting activity and ability to generate oxidative stress.^54–59^ In this case, the relationship between exposure to the above antibiotic groups and an increased secretion of EVs is associated with the activation of prophages^56^ or the mechanism of selective removal of oxidized proteins from microbial cells.^58^ Interestingly, *H. pylori* has been shown to respond to oxidative stress by secreting numerous catalase-containing EVs, which promoted the survival rate of this bacterium under these conditions.^31,60^ The data describing the influence of translation-inhibiting antibiotics (e.g. macrolides such as clarithromycin) on the production of EVs are very limited; however, it has been indicated that due to their bacteriostatic activity an effect on the number of these structures is marginal.^54,61^

In addition to the above, we observed that the level of EV secretion is strain-dependent, being highest for *H. pylori* 3CML (∼3–6 × 10^11^ particles/mL) and lowest for *H. pylori* 2CML (∼3 × 10^9^ particles/mL) (Figure 4o and h). However, it should be noted that the size distribution of EVs generated by *H. pylori* 2CML is drastically different from that observed for the other two strains. A careful inspection of the size distributions allowed us to see the existence of a second fraction of EVs with dimensions of 400–700 nm (Figure 4k–n). In our opinion, this may result from the unique features of EVs from *H. pylori* 2CML, contributing to their autoaggregation and a significant increase in large-sized EVs at the expense of reducing their total concentration. Similar observations regarding EVs of *H. pylori* NCTC11639 were made by the team of Grande *et al*.^43^

The modulatory effect of sub-MICs of antibiotics on the other two parameters of EVs, mean size and electrical charge, was variable and strain-dependent (Figure 4b, c, i, j, p and q). Currently we are unable to determine the cause of this phenomenon, although we suspect that the translation-inhibiting activity of clarithromycin^62^ and nucleic acid–targeting action of both metronidazole and levofloxacin^63,64^ may lead to quantitative changes in the proteinaceous and extracellular DNA cargo of EVs. These changes may in turn induce intricate modifications in physicochemical parameters of these structures. Deepening the background of this phenomenon and its effect on biological functions of EVs is undoubtedly crucial in the future.

### Fatty acid profiles of EVs

Recently published studies by our team^33^ and others^65^ have shown that antibiotic stress in *H. pylori* contributes to changes in the biosynthesis of membrane fatty acids and leads to modifications in the ability of this bacterium to produce biofilms. Considering the above observations and the fact that the most important components of EVs are lipids,^66^ in the last stage of our studies we decided to verify how sub-MICs of antibiotics affect the profile of membrane fatty acids of *H. pylori* EVs.

**Figure 5.**
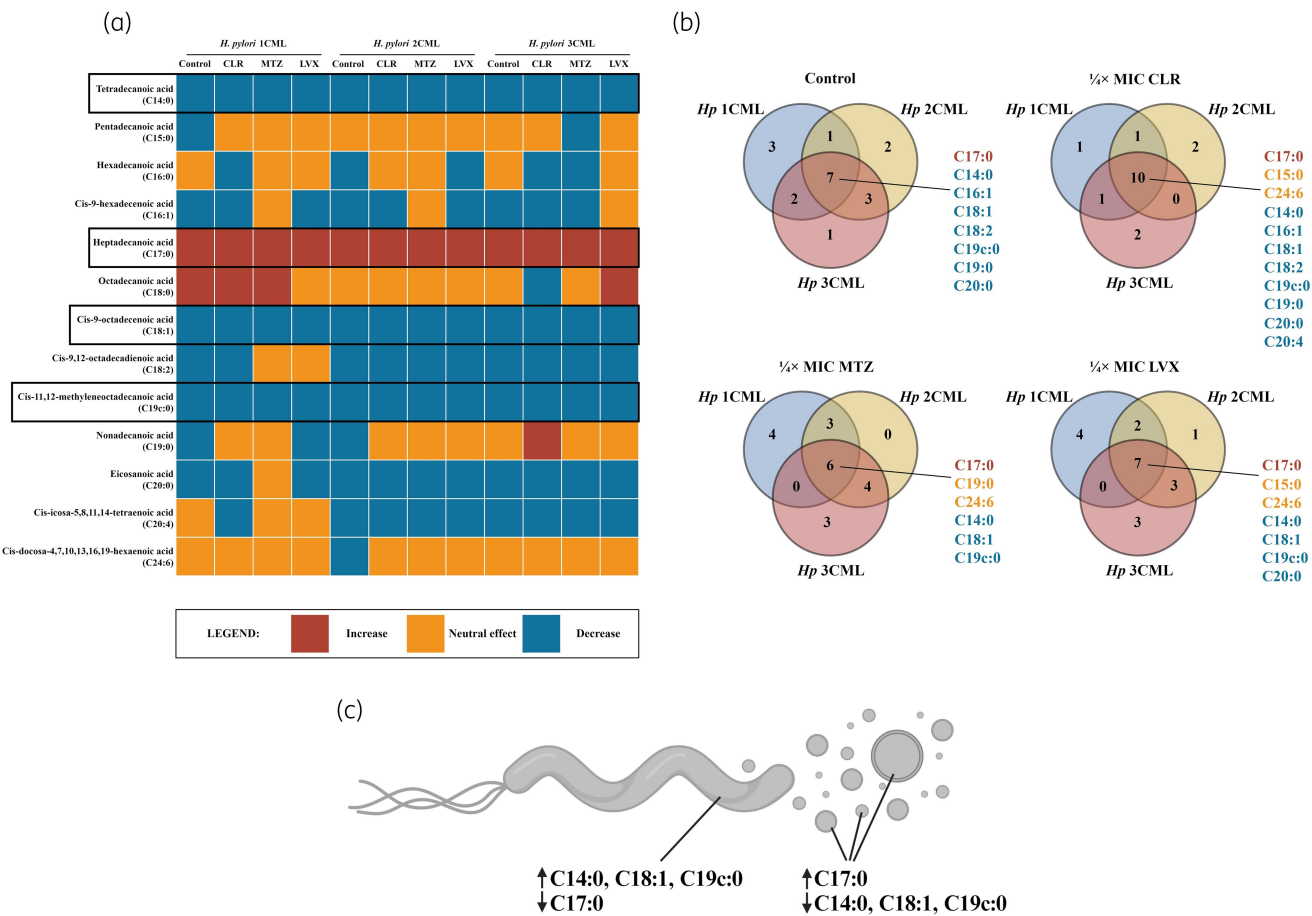
Assessment of changes in the fatty acid profile of extracellular vesicles (EVs) of clinical *H. pylori* strains cultured under antibiotic stress. Bacteria were incubated in BHI + 10% FCS^-EVs^ in 24-well titration plates for 3 days at 37°C under microaerophilic conditions and shaking at 100 rpm. The EV isolation procedure is shown in Figure 3. The content of individual fatty acids in the analysed samples was determined by comparing the ratio of the signal intensity of the analyte to the signal intensity of the internal standard used. The results obtained for EVs were normalized to measurements of bacterial cells and ratios of ≥2 (increase) or ≤0.5 (decrease) were considered significant. (a) Heat map showing changes in the profile of fatty acids constituting EVs of *H. pylori* strains. (b) Venn diagrams showing common features in the EV fatty acid profiles of *H. pylori* strains. (c) Graphic proposing the involvement of EVs in maintaining the appropriate fatty acid composition of cell membranes of *H. pylori*. CLR, clarithromycin; *Hp*, *Helicobacter pylori*; LVX, levofloxacin; MTZ, metronidazole. Part (c) was created in bioRender (https://BioRender.com/e92z925).

Based on knowledge of the most important fatty acid components of *H. pylori* cell membranes,^33,67,68^ we focused our current research on a detailed analysis of the quantitative changes in 13 representatives of this group (Figure 5). By normalizing the EV fatty acid profiles to those of bacterial cells, we obtained information regarding the selective packaging of specific fatty acids into EVs or their selective retention in bacterial cell membranes. The heat map indicates antibiotic- and strain-dependent modifications in the EV fatty acid profiles (Figure 5a). Nevertheless, a careful examination of these results showed the existence of a conservative pattern for the four fatty acids tested. Regardless of the conditions and the strain used, we observed a significant decrease in tetradecanoic acid (C14:0; 5–52-fold ↓), *cis*-9-octadecenoic acid (C18:1; 4–47-fold ↓) and *cis*-11,12-methyleneoctadecanoic acid (C19c:0; 23–233-fold ↓) in EVs, with a simultaneous substantial increase in heptadecanoic acid (C17:0; 5–64-fold ↑) (Figure 5a and c). It is worth noting that C14:0, C18:1 and C19c:0 are the most important fatty acids constituting *H. pylori* cell membranes, whereas the level of C17:0 in membranes of these bacteria is marginal.^33,67,68^ On this basis, we conclude that the selective packaging of C17:0 into EVs and the firm restriction of this process for the remaining three fatty acids may represent a mechanism of cell membrane homeostasis and rearrangement that has not yet been described for *H. pylori*. For other bacteria, the ability to selectively package misfolded proteins,^58,69^ damaged membrane components^70^ or unfavourable lipopolysaccharide forms^71–73^ into EVs as a mechanism for dynamic adaptation to changing environmental conditions has been demonstrated.

Additionally, our results were presented in the form of Venn diagrams to allow for a better insight into shared features of all *H. pylori* strains during their response to antibiotic stress (Figure 5b). Taking into account only fatty acids undergoing significant modifications, we observed a high convergence in EV profiles between the control and ¼ × MIC of clarithromycin, as well as between ¼ × MIC of metronidazole and ¼ × MIC of levofloxacin. This suggests that the type of antibiotic influences the intensity of membrane fatty acid remodelling and, unlike clarithromycin, exposure to metronidazole or levofloxacin promoted more drastic changes in the EV fatty acid profile. These results are consistent with our previous findings highlighting that exposure of *H. pylori* to ¼ × MIC of metronidazole or levofloxacin was accompanied by modifications in its membrane fatty acid profile towards a pattern typical for biofilms, without such adaptation during treatment with ¼ × MIC of clarithromycin.^33^ Interestingly, in *Pseudomonas* spp. it has been shown that the transition of bacteria to the stationary phase or their exposure to environmental stress is associated with EV release–dependent changes in the biophysicochemical parameters of bacterial cell membranes and promotion of biofilm development.^74,75^ In line with the information above, we believe that a strong correlation between the EV secretion and biofilm development of *H. pylori* may exist, as proposed in studies by different teams.^32,43,45,46^

### Research implications and future directions

On the one hand, our current research indicates the involvement of vesiculation in adaptative responses of *H. pylori* against environmental challenges, including the presence of antimicrobials. According to literature data, antibiotic stress enhances fatty acid biosynthesis in *H. pylori*, whereas inhibition of this process reduces the ability of this bacterium to produce biofilm and potentiates the activity of antibiotics.^65,76^ Our current results complement the above data by showing that EVs are involved in modulating the lipid profile of *H. pylori* cell membranes during the antibiotic exposure. This fact allows us to conclude that the secretion of EVs by *H. pylori* constitutes an interesting target for future therapies that could sensitize this microorganism to drugs.

On the other hand, it should be noted that EVs of *H. pylori* may carry many virulence factors (toxins or lytic enzymes) and therefore play a key role in the development of gastric and extragastric diseases.^26–30^ Considering the results of our present studies indicating that antibiotics can affect parameters of EVs produced by *H. pylori*, including their number or dimensions, it seems reasonable to verify how these modifications affect the cytotoxicity of EVs towards host cells. Undoubtedly, future determination of the impact of antibiotics on the course of EV-dependent *H. pylori* infections is another valuable research path.

### Summary

In conclusion, our data demonstrate that antibiotic stress in *H. pylori* affects the secretion intensity and physicochemical features of EVs produced by this bacterium in a strain- and antibiotic-dependent manner. Additionally, we show that EVs of *H. pylori* participate in the rearrangement of the bacterial cell membrane fatty acids and therefore may represent an interesting target for future therapies.

## Supplementary Material

dkaf172_Supplementary_Data

## Supplementary data

Figures S1 and S2 and Table S1 are available as Supplementary data at *JAC* Online.
