# Supplementary material for: Antibiotic stress affects the secretion and physicochemical features of extracellular vesicles produced by Helicobacter pylori
Source: J Antimicrob Chemother. 2025 May 29;80(7):2032–43. doi: 10.1093/jac/dkaf172 (PMC12209803; doi:10.1093/jac/dkaf172)
Supplement: dkaf172_Supplementary_Data [file dkaf172_supplementary_data.zip › Impact of antibiotics on EVs of H. pylori - supplementary materials.docx]

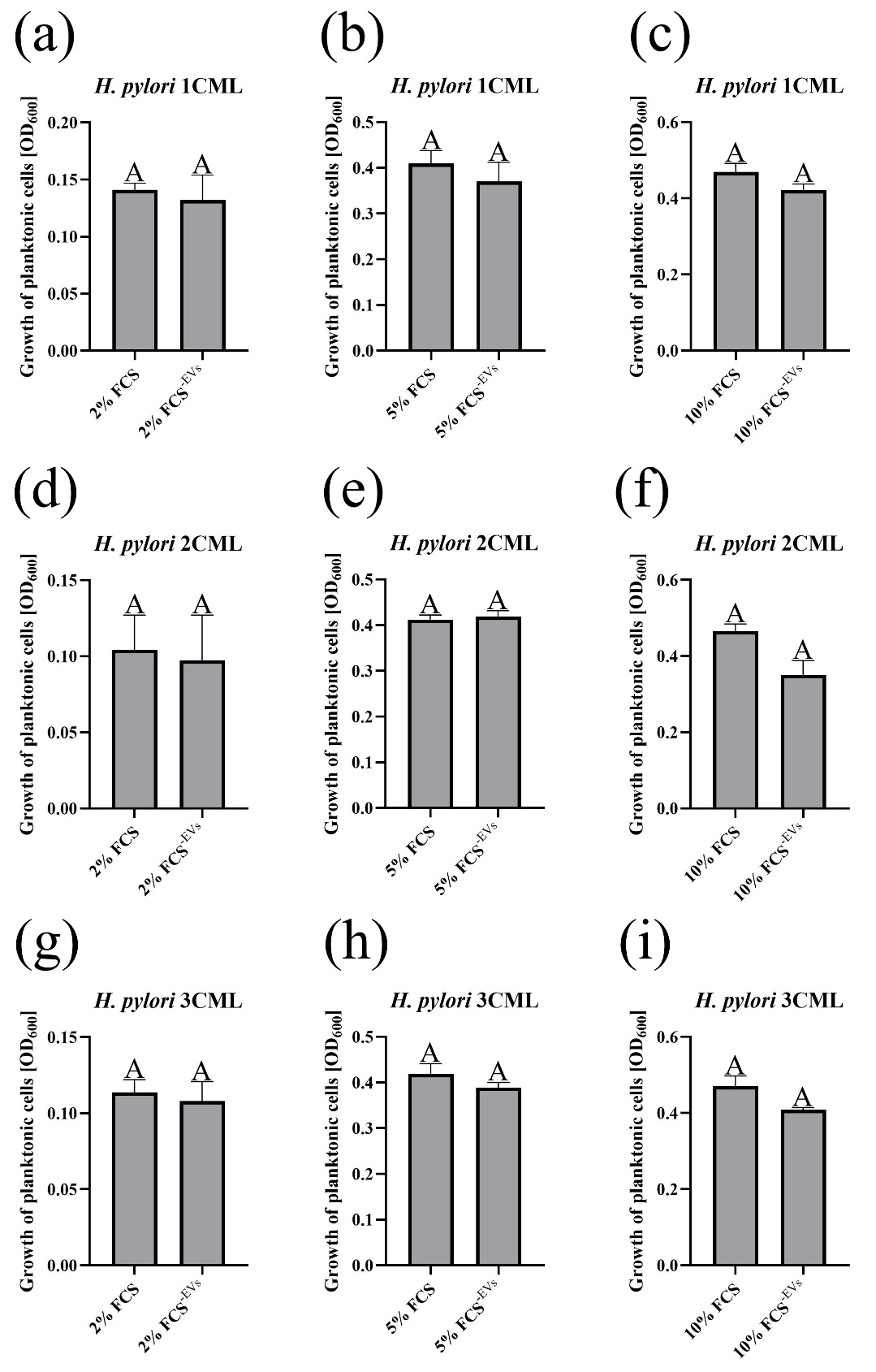


**Figure S1.** Assessment of the planktonic cells growth of clinical *H. pylori* strains cultured in BHI supplemented with various concentrations of classic (FCS) and EVs-depleted (FCS^-EVs^) serum. Bacteria were incubated in 24-well titration plates by 3-day at 37°C, microaerophilic conditions and shaking at 100 rpm. The growth of planktonic forms was evaluated spectrophotometrically. The growth of *H. pylori* 1CML in BHI with 2% (a), 5% (b) and 10% (c) serum. The growth of *H. pylori* 2CML in BHI with 2% (d), 5% (e) and 10% (f) serum. The growth of *H. pylori* 3CML in BHI with 2% (g), 5% (h) and 10% (i) serum. Each value is representative of three biological replications (mean ± standard error of the mean). The values with different subscript letters in a column are significantly different (p < 0.05, Kruskal–Wallis test with Holm correction).

**
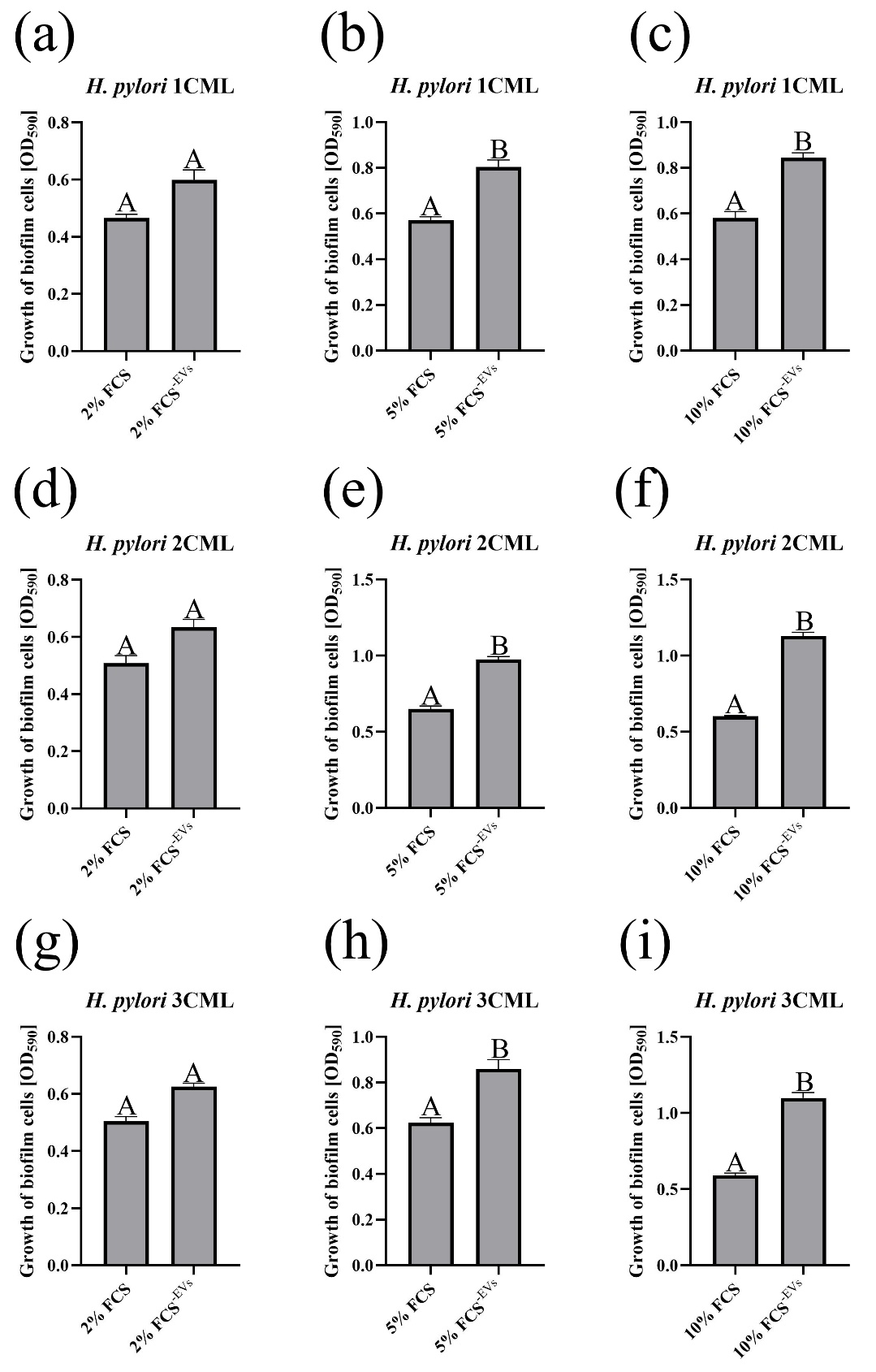
**

**Figure S2.** Assessment of the biofilm cells growth of clinical *H. pylori* strains cultured in BHI supplemented with various concentrations of classic (FCS) and EVs-depleted (FCS^-EVs^) serum. Bacteria were incubated in 24-well titration plates by 3-day at 37°C, microaerophilic conditions and shaking at 100 rpm. The growth of biofilm forms was determined using a crystal violet staining assay and spectrophotometry. The growth of *H. pylori* 1CML in BHI with 2% (a), 5% (b) and 10% (c) serum. The growth of *H. pylori* 2CML in BHI with 2% (d), 5% (e) and 10% (f) serum. The growth of *H. pylori* 3CML in BHI with 2% (g), 5% (h) and 10% (i) serum. Each value is representative of three biological replications (mean ± standard error of the mean). The values with different subscript letters in a column are significantly different (p < 0.05, Kruskal–Wallis test with Holm correction).

**Table S1.** MICs of antibiotics ​​for tested *H. pylori* strains in BHI broth with 10% FCS^-EVs^.

| **Tested stains** | **MICs [mg/L]** | | |
| --- | --- | --- | --- |
|  | **Clarithromycin** | **Metronidazole** | **Levofloxacin** |
| *H. pylori* 1CML | 8 | 256 | 64 |
| *H. pylori* 2CML | 16 | 256 | 64 |
| *H. pylori* 3CML | 8 | 512 | 64 |
