# Supplementary figures and images for: Antibiotic stress affects the secretion and physicochemical features of extracellular vesicles produced by Helicobacter pylori
Source: J Antimicrob Chemother. 2025 May 29;80(7):2032–43. doi: 10.1093/jac/dkaf172 (PMC12209803; doi:10.1093/jac/dkaf172)

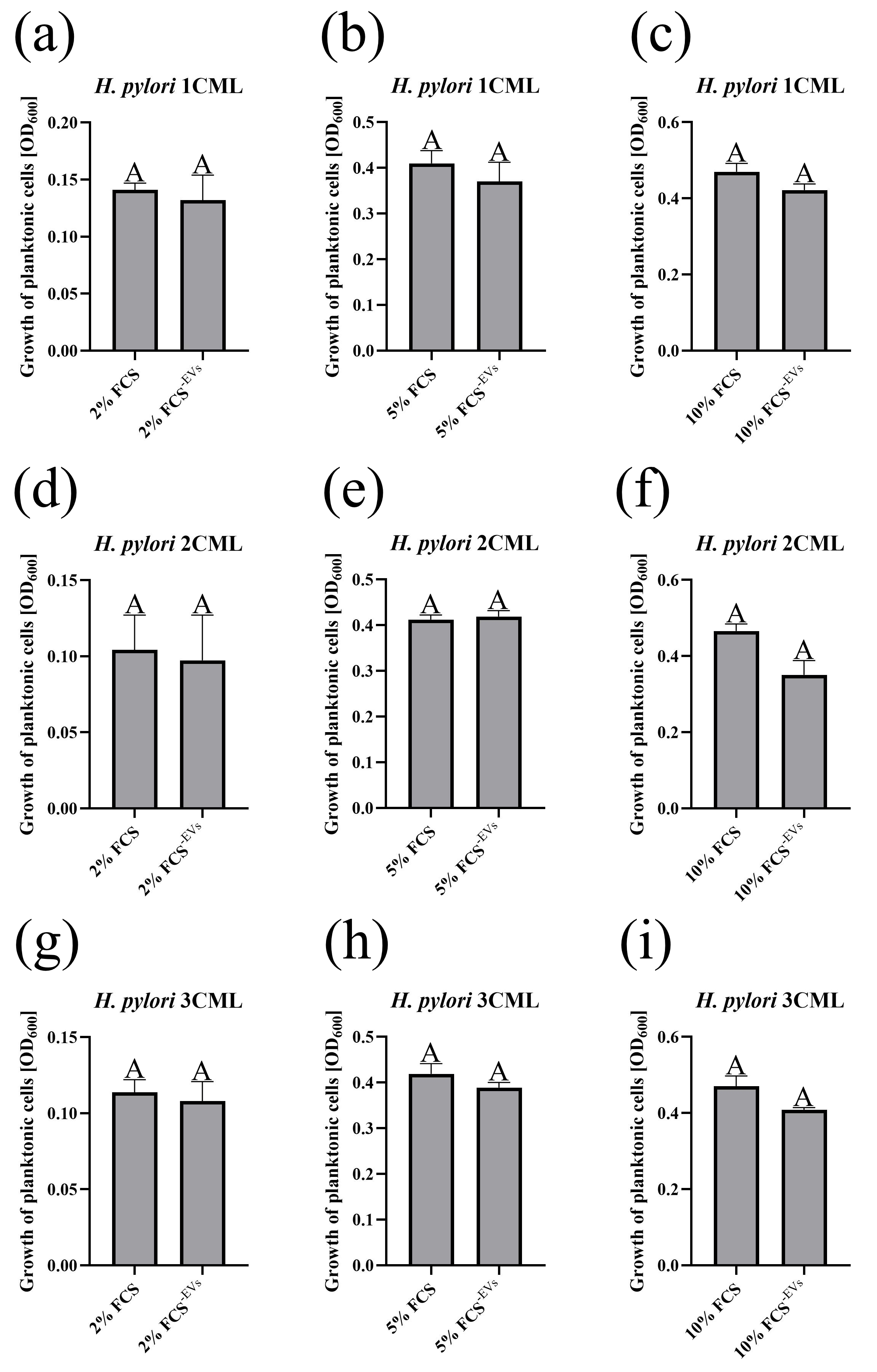

Supplement: dkaf172_Supplementary_Data [file dkaf172_supplementary_data.zip › Figure_S1.jpg]

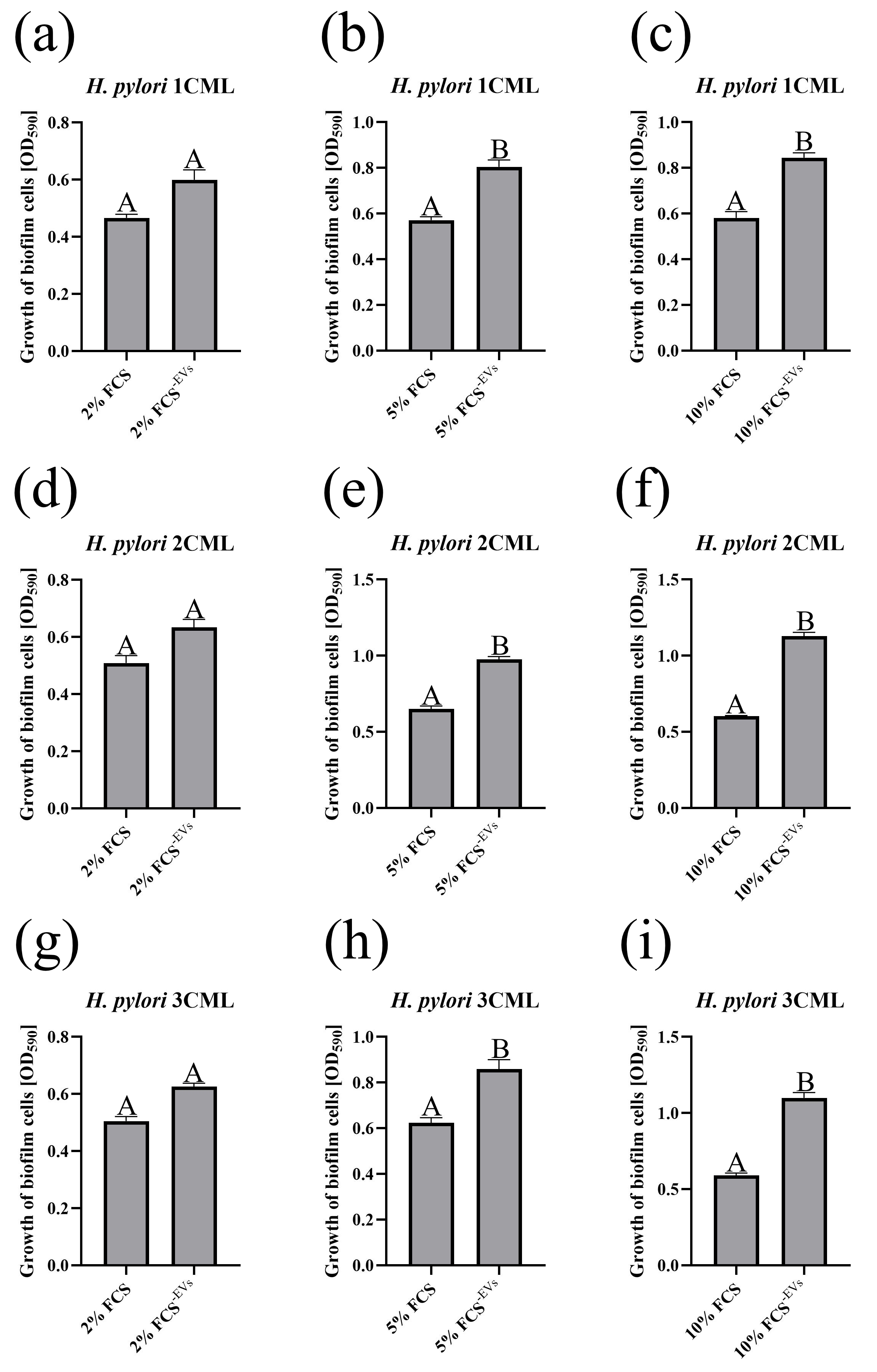

Supplement: dkaf172_Supplementary_Data [file dkaf172_supplementary_data.zip › Figure_S2.jpg]
